# Structure-Function Relationships of Covalent and Non-Covalent BTK Inhibitors

**DOI:** 10.3389/fimmu.2021.694853

**Published:** 2021-07-19

**Authors:** Rula Zain, Mauno Vihinen

**Affiliations:** ^1^ Department of Laboratory Medicine, Clinical Research Centre, Karolinska Institutet, Karolinska University Hospital, Huddinge, Sweden; ^2^ Centre for Rare Diseases, Department of Clinical Genetics, Karolinska University Hospital, Stockholm, Sweden; ^3^ Department of Experimental Medical Science, Lund University, Lund, Sweden

**Keywords:** BTK inhibitors, ibrutinib, acalabrutinib, zanubrutinib, fenebrutinib, protein-inhibitor interactions, covalent and non-covalent binding, structure-function relationship

## Abstract

Low-molecular weight chemical compounds have a longstanding history as drugs. Target specificity and binding efficiency represent major obstacles for small molecules to become clinically relevant. Protein kinases are attractive cellular targets; however, they are challenging because they present one of the largest protein families and share structural similarities. Bruton tyrosine kinase (BTK), a cytoplasmic protein tyrosine kinase, has received much attention as a promising target for the treatment of B-cell malignancies and more recently autoimmune and inflammatory diseases. Here we describe the structural properties and binding modes of small-molecule BTK inhibitors, including irreversible and reversible inhibitors. Covalently binding compounds, such as ibrutinib, acalabrutinib and zanubrutinib, are discussed along with non-covalent inhibitors fenebrutinib and RN486. The focus of this review is on structure-function relationships.

## Introduction to Bruton Tyrosine Kinase (BTK)

The B-cell-receptor (BCR) signaling pathway includes several components among which Bruton tyrosine kinase (BTK) is one of the key players (1, 2). The B-cell surface receptor is activated upon binding of a ligand (antigen). This in turn initiates a cascade of intracellular signaling events in which BTK is involved. BTK belongs to protein tyrosine kinases (PTKs), which transfer the gamma phosphate of adenosine triphosphate (ATP) to specific tyrosine residues in the target proteins (3, 4). BTK is an essential signaling molecule for the development, differentiation and survival of B-cells (2, 5). It is expressed in the majority of the cells of the hematopoietic lineage, except for the T- and plasma cells (6, 7).

BTK is encoded by the *BTK* gene (2, 5) and is a member of the Tec family of PTKs along with BMX non-receptor tyrosine kinase (BMX), IL2 inducible T cell kinase (ITK), tec protein tyrosine kinase (TEC), and TXK tyrosine kinase (TXK). Apart from TXK, they all have five structural domains. In the N-terminus, pleckstrin homology (PH) domain, which has a membrane-localizing function, is followed by the Tec homology (TH) region, which is unique for the Tec family. PH domain is a versatile docking domain that has numerous binding partners. The PH domain and BTK motif are missing in TXK. Between the TH region and the C-terminus there are the Src homology 3 (SH3), SH2 and kinase domains. The SH2 and SH3 domains have binding functions, whereas the kinase domain catalyzes the phosphorylation of tyrosine residues in target molecules.

PTKs are key components in cellular signal transduction pathways. Their activity is tightly controlled. This is crucial since they regulate a variety of cellular functions, such as cell growth, differentiation and malignant transformation. Gain-of-function variants activate signalling pathways even in situations when they should be silenced. Such active pathways are harmful and are related e.g. to various forms of cancer (8).

The human kinome consists of 518 protein kinases (including atypical ones) (9), among which BTK contains the largest number of different disease-causing variations. Variations in BTK can, for example, lead to a rare primary immunodeficiency called X-linked agammaglobulinemia (XLA) (XLA, MIM #300755) (5), which is characterized by low B-cell numbers and lack of immunoglobulins leading mainly to bacterial infections in patients (10). Antibody substitution therapy is an efficient treatment but requires lifetime management. XLA is caused by loss-of-function variations. Variations in BCR signalling pathway can lead to constitutive signalling and are common in B cell malignancies. Gain-of-function variants have been identified e.g. in Waldenström macroglobulinemia (11) and in chronic lymphocytic leukemia (CLL, MIM #151400) (12) and in some other B cell malignancies, for which there are efficient treatments (13, 14). BTK gain-of-function variants have been studied in the laboratory (15, 16).

## BTK Protein Structure

**Figure 1:**
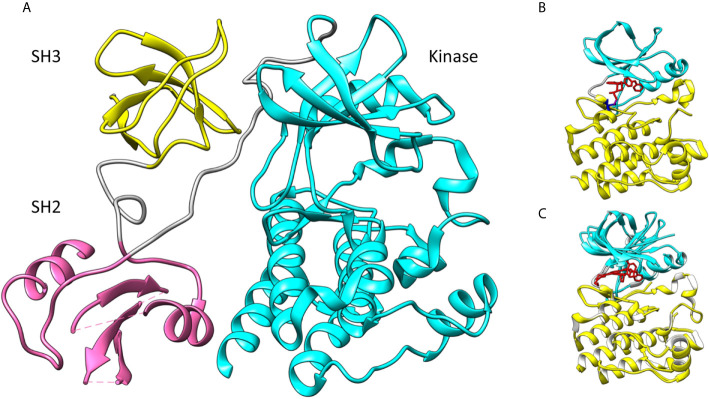
**(A)** Spatial organization of mouse BTK SH3 (yellow), SH2 (pink) and kinase domains (cyan). Linkers are in gray. The SH2 and SH3 domains are indicated by their positions in the dimer (PDB id 4xi2). **(B)** Human BTK kinase domain with ibrutinib (red) bound covalently (magenta) to C481 (blue) (5p9j). The upper lobe is in cyan, the lower lobe in yellow and inhibitor in red. **(C)** Superimposition of closed (5p9j) and open BTK kinase conformation (1k2p). Upper lobe is in cyan and lower lobe in yellow. Lower lobe was used for the superimposition.

In this review we concentrate on the BTK kinase domain. It is the only catalytic part in the Tec family PTKs. It consists of about 250 residues and is connected to the preceding SH2 domain by a long linker. **Figure 1A** shows the overall arrangement of the SH3 (yellow in the figure), SH2 (pink) and kinase domains (cyan) in mouse BTK (PDB id 4xi2) (17) for which there is the longest experimentally defined structure in the Tec family. The full structure with all domains has not been determined for any protein kinase. The overall structures of PTKs are flexible. Kinase domains can appear in active and inactive conformations. In the case of BTK, an elongated structure has been determined with small-angle X-ray scattering (SAXS) (18), however, there are likely several different conformations depending e.g. on the phosphorylation status and interactions between domains and with other proteins and compounds.

The kinase-domain scaffolding in PTKs is formed of two lobes and the catalytic site is between the lobes (**Figure 1B**) (PDB id 5p9j) (19). The smaller N-terminal lobe contains antiparallel β-sheets and one or two α-helices. The lower, C-terminal lobe usually has seven helices and some short β-strands. ATP that provides the phosphate group for the transferase reaction is bound in a cleft between the two lobes. This site is occupied by covalent inhibitors. Ibrutinib binding to BTK is shown in **Figure 1B**. Orientations of the two lobes vary. When the enzyme is in the active, also called a closed, conformation the lobes form ATP and ligand binding regions. In the inactive form, the enzyme has an open conformation and cannot effectively bind to ATP, the cofactor of the reaction. **Figure 1C** indicates how the BTK upper lobe turns while the lower lobe remains largely similar in the conformations for closed structure with ibrutinib (5p9j) (19) and open form (1k2p) (20).

The upper lobe rotates in relation to the lower lobe during activation. Phosphorylation of a tyrosine residue in the activation loop (Y551 in BTK) is required for activity. Structural changes during activation include rotation of the upper lobe to lock the ATP molecule between the two domains. During this process, some reorganization of secondary structural elements and loops happens. Following BCR engagement, BTK translocates to the plasma membrane with the help of the PH domain. It leads to Y551 phosphorylation by a Src family kinase, mainly LYN.

The flexibility and dynamics of the kinase domain is facilitated by the linker between the lobes (**Figure 1C**). A single polypeptide chain connects the two lobes and minor changes in this flexible region lead to different relative orientations of the lobes. Depending on the binding ligand, e.g. an inhibitor, the angle between the domains varies implying a dynamic “rigid body” movement, where the entire lobes are turned in relation to each other.

### ATP-Binding Pocket

**Figure 2:**
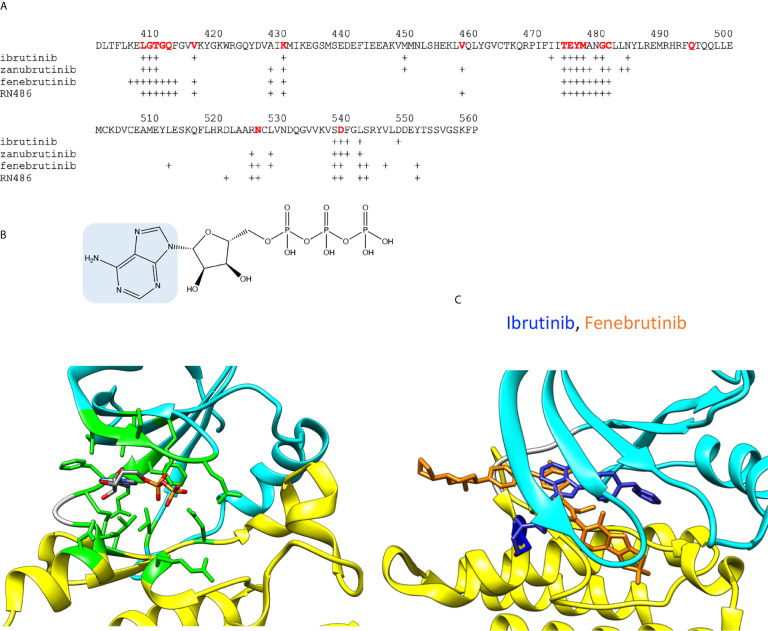
ADP and inhibitor binding. **(A)** BTK sequence indicating amino acids within 5 Å radii from ADP or inhibitors. For ATP binding, residues corresponding to interacting amino acids in BTK are shown in red. Crosses indicate the interacting amino acids in inhibitor complexes. **(B)** *Upper panel*: Chemical structure of ATP, adenine is highlighted in blue. *Lower panel*: ADP bound to ITK kinase domain (4m15). Residues within 5 Å from ADP are in green. Atoms in the ADP are colored based on the elements, carbon gray, nitrogen blue, oxygen red, and phosphorus orange. **(C)** Differences in the binding modes of the covalent inhibitor, ibrutinib (blue, 5p9j), and non-covalent inhibitor, fenebrutinib (orange, 5vfi), to BTK. C481 is in blue.

The overall sequence similarity of PTKs is rather low, still they show conserved protein folds. The ATP binding site is among the most conserved regions. **Figures 2A, B** show the amino acids involved in ATP binding in BTK based on the corresponding residues when ADP is bound to ITK (4m15) (21). Amino acids within 5 Å distance from the cofactor were identified. The binding site is formed by amino acids folding together from different parts of the protein, within a stretch of about 140 amino acids. The majority of the PTK targeting drugs have been designed to intervene ATP binding and thereby inhibiting the catalytic activity. There are enough differences between PTKs to facilitate the development of rather specific inhibitors. Some other inhibitors have been designed to target outside of the ATP binding pocket.

Although the ATP binding sites are conserved in PTKs, each one shows unique features. The ATP-binding site holds the terminal phosphate group in a position in relation to the catalytic residue D521 and the ligand to be phosphorylated. The phosphorylation reaction requires a complex structure and many interactions. The substrate recognition region interacts with the ligand and holds it in a proper orientation in relation to ATP and the catalytic residues, which again are oriented by an extensive and intricate network of interactions including proper orientation of the kinase lobes and a Mg^2+^ ion essential for the catalysis (22).

A large group of PTK inhibitors have been designed to occupy the ATP-binding pocket, and therefore they can be considered as “ATP-like” compounds partially sharing a similar chemical structure with adenine (**Figure 2B**). These inhibitors form hydrogen bonds with the amino acid residues in the region between the two kinase lobes (23, 24). The corresponding H-bonds are naturally formed by the endo- and exocyclic amino groups of adenine in ATP. The selectivity of the inhibitors can be conferred through extended interactions with the surrounding regions including those, otherwise, occupied by the ribose moiety, the hydrophobic part and the triphosphate group of ATP.

## BTK Inhibitors

The number of chemical compounds developed to inhibit BTK activity is continuously increasing, and three compounds are already approved as drugs by the U.S. Food and Drug Administration (FDA) and the European Medicine Agency (EMA). These are ibrutinib, acalabrutinib and zanubrutinib (**Table 1**) (25). The majority of BTK inhibitors target the ATP-binding site and they are classified into two categories based on their binding mode as reversible or irreversible inhibitors. For additional recent reviews on BTK inhibitors see (26, 27) for the chemical point of view and the development of BTK inhibitors in cancers, respectively.

**Table 1:** Examples of reversible and irreversible BTK inhibitors.

Inhibitor	Molecular weight (g/mol)	PDB ID^a^	Mode of binding
Ibrutinib	440.5	5p9j	*covalent* irreversible
Acalabrutinib	465.5	–	*covalent* irreversible
Zanubrutinib	471.5	6j6m	*covalent* irreversible
Fenebrutinib	664.8	5vfi	*non-covalent* reversible
RN486	606.7	5p9g	*non-covalent* reversible
LOXO 305	479.4	–	*non-covalent* reversible
BMS-986142	572.6	–	*non-covalent* reversible
Rilzabrutinib	665.8	–	*covalent* reversible

a ID for the entry used in the analysis and text.

**Figure 3:**
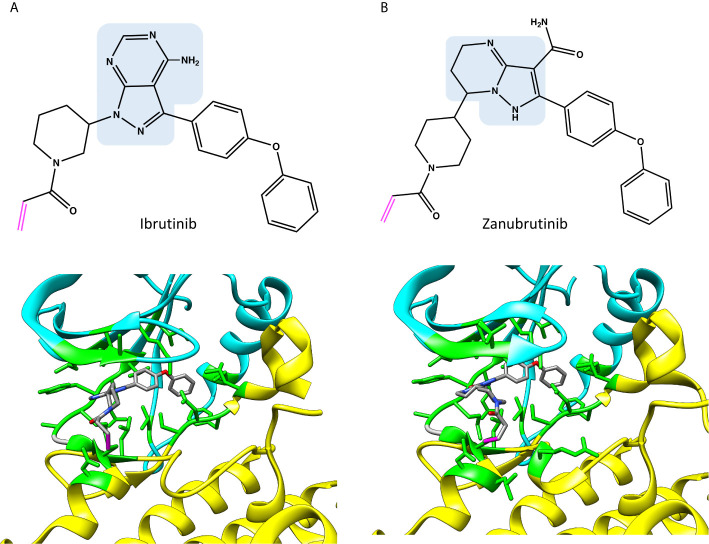
Chemical structures and binding of covalent BTK inhibitors, **(A)** ibrutinib (5p9j), and **(B)** zanubrutinib (6j6m). Residues within 5 Å distance from inhibitor are shown in green. Atoms in the inhibitors are colored based on the elements, carbon gray, nitrogen blue, oxygen red. Covalent bond between C481 and inhibitor is in magenta. The structures have the same pose as in **Figure 2C**.

Despite markedly different binding modes of irreversible and reversible inhibitors, they share many common interacting residues. Covalent inhibitor ibrutinib (5p9j) (19, 28) and non-covalent inhibitor fenebrutinib (5vfi) (29) represent the two classes in **Figure 2C**. Residues around 410, 430, 480 and 540 (**Figure 2A**) are involved in interactions with both types of inhibitors. In addition, there are group- and inhibitor-specific interactions. The covalent inhibitors ibrutinib and zanubrutinib (**Figure 3**) have slightly smaller number of possible interactions (19 and 22, respectively) than the non-covalent drugs (**Figure 5**) fenebrutinib and RN486 (29 and 26, respectively). This could be explained by the size of the molecules and by the fact that non-covalent inhibitors may need more interactions to achieve the affinity required from a drug. These inhibitors, from both categories, are discussed in the following sections.

### Irreversible Inhibitors

Protein inhibitors can be equipped with a reactive functional group, which is able to form a covalent bond with an amino acid residue. One major advantage of covalent kinase inhibitors is that high selectivity can be obtained through a combination of both non-covalent and covalent interactions. Side chains in both cysteine and serine are frequently targeted by irreversible kinase inhibitors. For example, the aliphatic thiol (SH) group within the side chain of cysteine, specifically in its deprotonated form (thiolate anion), is an excellent nucleophile, which can react with an electrophilic functional group in the inhibitor designed specifically to form a stable covalent bond.

The ATP binding site in BTK includes a cysteine (C481), which has been examined as a target for several irreversible inhibitors. This residue is not highly conserved in PTKs, but several PTKs have been targeted at the corresponding amino acid. Sequence alignment of BTK and Src family kinases and screening of first-generation inhibitors that bind to the ATP-site revealed that C481 in BTK could act as a nucleophile and form a covalent bond with an inhibitor (30). This led to the birth of ibrutinib, the first irreversible BTK inhibitor, which is approved as treatment for several B-cell malignancies (25, 31).

**Figure 4:**
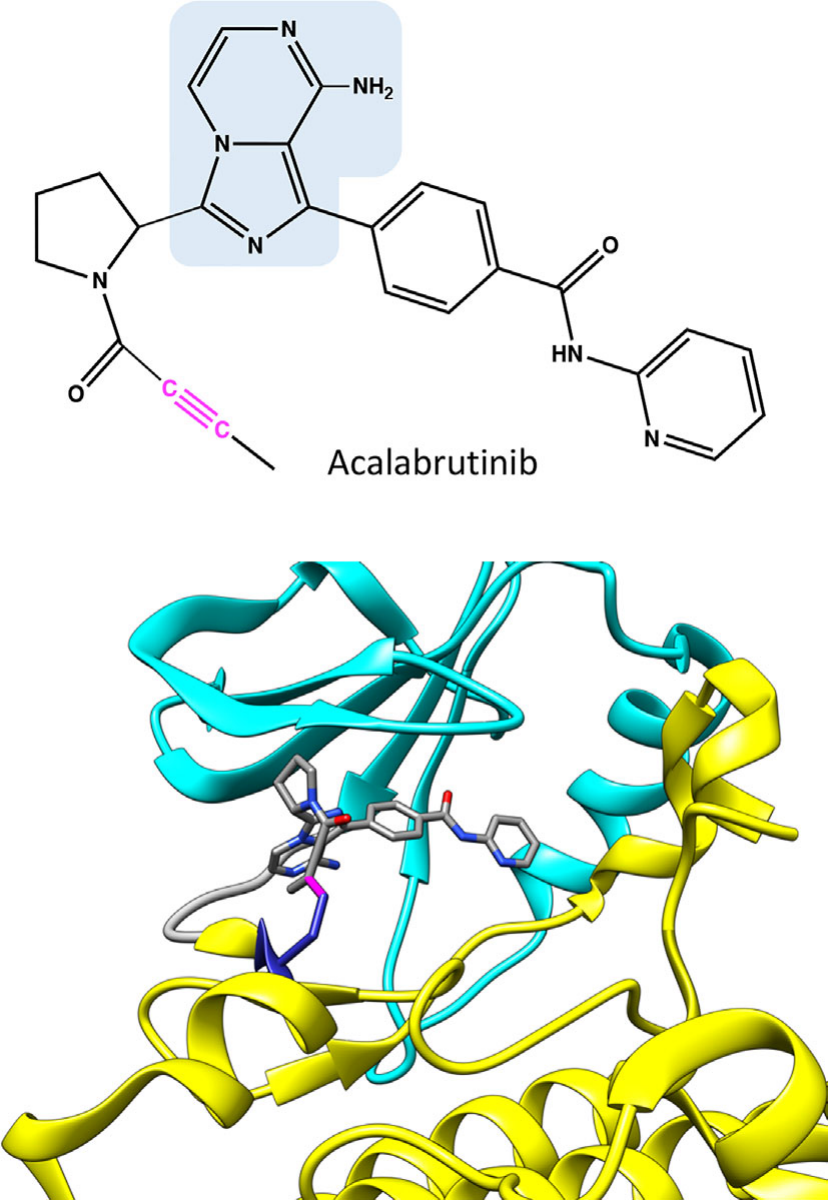
Chemical structure of acalabrutinib and molecular model showing binding of the covalent inhibitor in BTK. Atoms in the inhibitor are colored based on the elements, carbon gray, nitrogen blue, and oxygen red. Covalent bond between C481 and inhibitor is in magenta. The structure has the same pose as in **Figure 2C**.

As **Figure 2A** indicates, many of the residues involved in ADP binding are essential also for inhibitor recognition. These data were obtained by recognizing amino acids within 5 Å distance from the inhibitors. This distance allows for minor adjustments in the structures as well as interactions mediated by solvent (water) molecules. Crystallographic structures have been determined both for ibrutinib and zanubrutinib (**Figure 3**), the corresponding complex for acalabrutinib has been modelled (**Figure 4**) (32). Backbone amide groups of E475 and M477 form hydrogen bonds with the pyrazolopyrimidine core, which extends towards T474 and the area close to α-C-helix (5p9j and 5kup) (19, 33). Residues E475 and M477 are involved in binding of all the presented inhibitors (**Figure 2A**).

Although ibrutinib binds efficiently to BTK, it also recognizes some other kinases. These off-targets have a cysteine within the ATP binding pocket, and hence bind irreversibly (34, 35). Ibrutinib binds to the other Tec family PTKs ITK, TEC, BMX and TXK (36, 37), as well as to BLK (BLK proto-oncogene, Src family tyrosine kinase), JAK3 (Janus kinase 3) (34, 35) and VGEFR2 (38).

Zanubrutinib (6j6m **Figure 3B**) is an efficient irreversible BTK inhibitor (39). The compound has some preference for BTK versus TEC and it does not inhibit ITK. Zanubrutinib forms H-bonds with the hinge region residues E475 and M477, similar to ibrutinib.

Acalabrutinib has a reactive butynamide group that can bind covalently to C481 in BTK (37) (**Figure 4**). The structural properties of this compound differ from ibrutinib, thereby it has decreased off-target binding; for example, epidermal growth factor receptor (EGFR) and ITK are not inhibited by acalabrutinib.

A drawback of irreversible inhibitors is that drug resistance in malignant diseases can develop when BTK variations at the catalytic site and the gatekeeper are not able to bind efficiently to irreversible inhibitors in treated patients (40, 41). This is a rather common event in patients treated with irreversible inhibitors and who have a relapse.

### Reversible Inhibitors

Many clinical and preclinical kinase inhibitors compete with ATP and bind in a non-covalent manner (**Table 1**). These compounds are recognized in a structure-based mode and achieve selectivity through recognition of unique features of target protein kinases. Moreover, the emergence of BTK variations at C481 or T474 gatekeeper position following treatment with irreversible inhibitors prompted efforts to design and synthesize new reversible kinase inhibitors.

**Figure 5:**
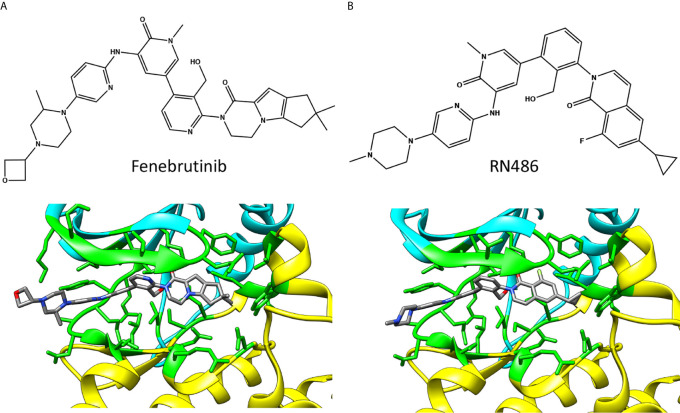
Chemical structures and BTK binding of non-covalent inhibitors **(A)** fenebrutinib (5vfi) and **(B)** RN486 (5p9g). Residues within 5 Å distance from inhibitor are shown in green. Atoms in the inhibitors are colored based on the elements, carbon gray, nitrogen blue, oxygen red, and fluorine pale green. The structures have the same pose as in **Figure 2C**.

Fenebrutinib (**Figure 5A**) was discovered through a structure-activity-relationship study of a series of precursor and analogue compounds (29). Fenebrutinib-BTK complex structure revealed specific interactions, which may explain its selectivity (25). This compound also showed retained activity *in vitro* towards BTK carrying the single and double variations C481S, T474A and T474S/C481S, respectively (32).

The reversible inhibitors interact with many amino acids that also interact with covalent inhibitors (**Figure 2A**) despite largely different binding orientation. M477 in BTK forms an H-bond with the pyridone oxygen in fenebrutinib and pendant NH group of the adjacent 2-aminopyridine (5vfi) whereas the pyridonyl ring packs against E408 and G480 (29).

In the search of new BTK reversible inhibitors with enhanced selectivity for the treatment of rheumatoid arthritis (RA), RN486 (**Figure 5B**) was developed and examined in *in vitro* binding and competition studies, as well as in cell-based assays (42). The compound showed efficient binding to BTK at subnanomolar concentration and exhibited anti-inflammatory effects in mice with collagen-induced arthritis (43). The structure of RN486-BTK complex (5p9g) (19) indicates quite similar binding mode of RN486 and fenebrutinib, see also **Figure 2A**.

Several additional small-molecules, which bind to BTK in a non-covalent manner, are currently under investigation, including LOXO-305 (44, 45) and BMS-986142 (46). An Interesting *covalent reversible* inhibitor is rilzabrutinib (PRN1008). The compound encompasses a functional nitrile group, which acts as the electrophile that reacts with the thiol of cysteine, thereby forming a covalent bond. The reactivity of this complex confers a possibility to reverse the reaction under *in vitro* and cellular conditions (47, 48). Pre-clinical studies of rilzabrutinib indicated anti-inflammatory effect in animal models of immune-mediated diseases (49).

### Inhibitor Binding and Selectivity

The binding affinity and activity of reversible and irreversible inhibitors are typically evaluated using *in vitro* biochemical and cellular assays, and are often translated into the value corresponding to the half-maximal inhibitory concentration (IC_50_) of the compound. IC_50_ can serve as a quantitative measure of the potency of reversible inhibitors. However, frequently used methods to estimate the binding and activity of kinase inhibitors in terms of IC_50_ values do not account for the two-step process of kinase inhibition by irreversible compounds. Furthermore, compound selectivity for one kinase over others are frequently determined based on IC_50_ values. However, these values differ depending on the assays used and in some cases lack kinetics measurements (25). Consequently, more accurate determination of the selectivity of irreversible inhibitors towards various kinases could be achieved through additional assays, such as the measurement of inactivation kinetics. This in turn may provide a better correlation with potential off-target activities and thereby explain certain adverse effects observed in treated patients (50, 51).

The two steps that govern the activity of irreversible inhibitors are binding affinity and formation of the covalent bond. The kinetics of the latter is a key parameter; however, not always included in the analysis and comparison of various BTK irreversible inhibitors. For example, in a recent study the relative selectivities were determined for the irreversible inhibitors ibrutinib and acalabrutinib towards BTK and TEC using both binding affinity and time-dependent activity (52). Drug selectivity was then evaluated in a cell-based occupancy assay in a Waldenström macroglobulinemia patient cell line and in CLL patient samples. In this system, ibrutinib and acalabrutinib showed comparable selectivity for BTK over TEC despite the fact that higher binding affinity, determined as IC_50_, was reported for acalabrutinib in BTK versus TEC (53, 54).

Further side-by-side comparisons of binding efficiency, kinetics and selectivity of BTK inhibitors in combination with structural profiling and molecular modeling would likely provide valuable pre-clinical predictions on the performance of both irreversible and reversible BTK inhibitors.

## Structure-Function Relationship (SFR)

XLA is caused by loss-of-function BTK variants. BTKbase, a database for BTK variants, was originally established 1994 (55) among the very first locus specific variation databases (LSDBs) and being the first one for primary immunodeficiencies. Currently, BTKbase contains information covering over 1800 individuals with 1928 DNA variants of which 985 are unique, originating totally from 1277 families (56).

**Figure 6:**
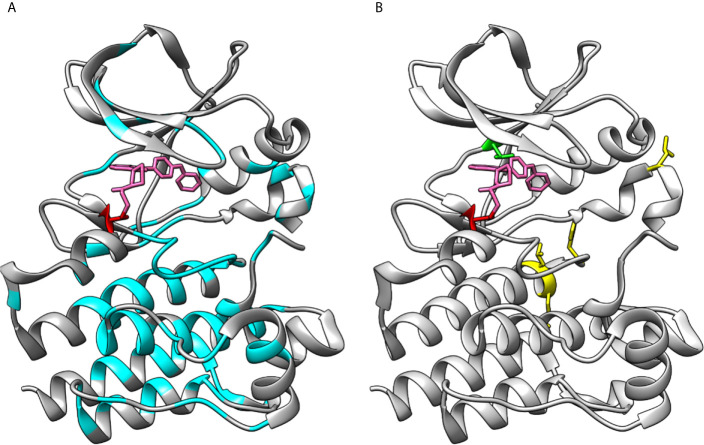
Loss- and gain-of-function variations in BTK kinase domain. **(A)** XLA-causing variants (cyan) are distributed all along the kinase domain. **(B)** Sites of ibrutinib resistance conferring amino acids C481 (red) and T474 (green) along with gain-of-function variants at positions 512, 513, 517 and 547 (yellow), note that variant at 474 alone and together with other variants lead to gain-of-function activities. The structures are for ibrutinib (pink) complex with BTK (5p9j).

In **Figure 6A** are shown the positions of XLA-causing variants in the kinase domain as reported in BTKbase. Similar to the other domains in BTK, disease-related variants are quite evenly distributed along the polypeptide chain (56). The presence of these variants can have several different biological consequences, whether of structural, functional, regulatory or other type. Some of them affect the ATP and ligand binding or catalytic or regulatory sites. Many variants likely alter the structure or function of the protein. Substitutions and splice site variants preventing protein expression are rather common. It has been predicted that about two thirds of the single nucleotide substitution-caused amino acid variations (SNAVs) and similar proportion of all possible single amino acid variants in the BTK kinase domain are likely harmful and disease-causing (57, 58). This is a relatively high proportion, however likely correct as there are many important sites and amino acids in the kinase domain.

Gain-of-function variants are rare in any protein and gene. Such variants in PTKs are harmful since the activity regulation mechanisms are not functional resulting in constitutive activation of the downstream signaling pathways (as far as the variant kinase is expressed and stable). Random mutagenesis screening for BTK gain-of-function variants revealed that single variants T474M and E513G, as well as T474 double variants with either L512M, E513G, F517L or L547P markedly increased the kinase activity (16). Apart from T474, these residues are located further away from the ATP binding site (**Figure 6B**) and the exact mechanism of increased activity is not known. The Catalogue Of Somatic Mutations In Cancer (COSMIC) does not contain known cancer variants of these types.

A gain-of-function variation, E41K, is known in the PH domain (15), thus activating variants can occur also in the other domains.

Variants at amino acids T474 and C481 lead to tolerance of covalent inhibitors in CLL and other B-cell malignancies. Effects of several variants and their combinations were recently investigated and shown to impair inhibitor binding leading to drug resistance (32). These two positions are indicated in **Figure 6B**.

### Structural Aspects of Adverse Effects

BTK inhibitors have been investigated in hundreds of clinical trials. Important part of these studies has been charting the adverse effects, which have been reported in detail elsewhere (25). The adverse effects vary among BTK inhibitors, and within the two major groups, reversible and irreversible inhibitors, as well as the treated diseases. Although bleedings, skin manifestations, diarrhoeas and cardiovascular diseases have been reported for certain covalently binding inhibitors, the molecular mechanisms causing these side effects are only starting to be discovered. Noteworthy, it is assumed that the adverse effects of covalent inhibitors are mainly due to off-target binding to other kinases that have cysteine in their binding site. On the other hand, ibrutinib, for example, binds also reversibly to additional kinases that do not have a cysteine in their binding pocket (35). These interactions inhibit additional pathways apparently with unwanted and harmful consequences.

In addition to the BCR signaling pathway, BTK is involved also in other signaling cascades initiated by Toll-like receptors or Fc-like receptors (59). A study of nine covalent and non-covalent BTK inhibitors indicated clearly different effects on these pathways (19). Covalent inhibitors as ibrutinib behaved differently from many reversible inhibitors. Thus, the reported adverse effects may emerge from the involvement of numerous pathways.

The positioning of Y551 and the surrounding activation loop differ markedly depending on the phosphorylation and on the ligand- or inhibitor binding status. Following Y551 phosphorylation, the activation loop is tightly bound to the structure, whereas in unphosphorylated form it is flexible and not even seen in many X-ray structures. RN486 and some other inhibitors change the loop conformation so that Y551 folds back to the protein, thereby “sequestering” it and making it solvent inaccessible (19). This effect is accompanied with differential outcomes in the signaling pathways and may contribute to different adverse effects.

## Concluding Remarks

BTK is a central signaling macromolecule in several pathways and variations in the BTK gene and protein may lead to the development of a range of diseases. Originally, it was identified as the causative protein for XLA due to loss-of-function variants. Subsequently it was implicated in B-cell malignancies, and more recently even in multiple sclerosis. The fact that several cancers are related to constitutive BCR signaling, including BTK activity, has rendered BTK as an important therapeutic target including small-molecule inhibitors. Numerous inhibitors have been developed and tested. First on the clinical practice were irreversible inhibitors that bind to the ATP binding site where C481 on the wall of this pocket forms an irreversible covalent bond with the inhibitor. More than 500 protein kinases are known in man and since all of them use ATP as the cofactor, the inhibitors are somewhat unspecific. Consequently, there is always some cross-reactivity, which can cause unwanted side effects (25). New compounds have been claimed to be significantly more specific than the first products examined and approved in the clinics. However, long-term clinical trials are not yet available for several new BTK inhibitors, including reversible compounds.

The residues C481 and T474 in BTK are both crucial for (irreversible) inhibitor interactions. Somatic variations in these sites lead to tolerance for irreversible inhibitors, which will no longer bind efficiently to the target site. These variants are quite common when cancer patients are treated with irreversible inhibitors. Reversible inhibitors, on the other hand, could be better in this respect as they form several specific interactions to achieve high enough affinity, and variation in one of these sites may not be detrimental for recognition.

## Author Contributions

RZ determined the general scope of the review. RZ and MV discussed and established the focus and details and drafted the manuscript. MV generated the BTK structures and RZ the chemical structures. All authors contributed to the article and approved the submitted version.

## Funding

Stockholm County Council, Swedish Medical Research Council and Swedish Cancer Society.

## Conflict of Interest

The authors declare that the research was conducted in the absence of any commercial or financial relationships that could be construed as a potential conflict of interest.
